# To the Editor of the New York Medical Journal

**Published:** 1867-01

**Authors:** Austin Flint


*To the Editor of the New York Medical Journal:*

DEAR SIR,—In the second edition of my work on the Principles and Practice of Medicine, I have inadvertently committed an error admitting of an inference which I beg permission through your columns to forestall. Giving some account of cholera in this city during the past summer, I have said as follows: "In New York, in anticipation of an epidemic visitation, the administration of affairs relating to public health was vested in four Commissioners, three of whom are distinguished members of the medical profession." (Page 473.)

It is an error of omission not to have stated that these four Commissioners acted in conjunction with the Police Commissioners and the Quarantine Physician; and inasmuch as there are four medical members in the Board, my language admits of the construction that only three of the four are distinguished members of the medical profession. To disclaim such a construction is the purpose of my asking you to insert this note. The quotation was penned under an impression, at the moment, that there were but three medical members in the Board. It is now too late to make the correction in the work, and I am therefore anxious, in justice to myself, to state that I had no intention of saying otherwise than that *all* the physicians in the Board of Commissioners are distinguised members of the medical profession. With much respect, very truly yours,

AUSTIN FLINT.

[*New York Med. Jour.*]

